# A Novel Tool for Visualization of Water Molecular Structure and Its Changes, Expressed on the Scale of Temperature Influence

**DOI:** 10.3390/molecules25092234

**Published:** 2020-05-09

**Authors:** Zoltan Kovacs, Bernhard Pollner, George Bazar, Jelena Muncan, Roumiana Tsenkova

**Affiliations:** 1 Department of Physics and Control, Faculty of Food Science, Szent István University, H-1118 Budapest, Hungary; 2 Department for Hygiene and Medical Microbiology, Medical University of Innsbruck, A-6020 Innsbruck, Austria; bernhard.pollner@me.com; 3 Department of Nutritional Science and Production Technology, Faculty of Agricultural and Environmental Sciences, Kaposvar University, H-7400 Kaposvar, Hungary; bazar@agrilab.hu; 4 Biomeasurement Technology Laboratory, Graduate School of Agricultural Science, Kobe University, Kobe 657-8501, Japan; jmuncan@people.kobe-u.ac.jp

**Keywords:** near infrared spectroscopy, chemometrics, aquaphotomics, water molecular mirror, aquagram

## Abstract

Aquaphotomics utilizes water-light interaction for in-depth exploration of water, its structure and role in aqueous and biologic systems. The aquagram, a major analytical tool of aquaphotomics, allows comparison of water molecular structures of different samples by comparing their respective absorbance spectral patterns. Temperature is the strongest perturbation of water changing almost all water species. To better interpret and understand spectral patterns, the objective of this work was to develop a novel, temperature-scaled aquagram that provides standardized information about changes in water molecular structure caused by solutes, with its effects translated to those which would have been caused by respective temperature changes. NIR spectra of Milli-Q water in the temperature range of 20–70 °C and aqueous solutions of potassium chloride in concentration range of 1 to 1000 mM were recorded to demonstrate the applicability of the proposed novel tool. The obtained results presented the influence of salt on the water molecular structure expressed as the equivalent effect of temperature in degrees of Celsius. The temperature-based aquagrams showed the well-known structure breaking and structure making effects of salts on water spectral pattern, for the first time presented in the terms of temperature influence on pure water. This new method enables comparison of spectral patterns providing a universal tool for evaluation of various bio-aqueous systems which can provide better insight into the system’s functionality.

## 1. Introduction

Recently, water has become more and more important subject of studies, as evidenced by the increasing number of water-related publications in various fields of science [1,2,3,4]. Water is fundamental to life—it is one of the essential and widely distributed components of biologic systems, which can be considered as a biomolecule in its own right [5,6]. Different scientific fields have been studying water from different aspects, trying to reach better understanding of its properties, structure and functions, yet still, our picture of water as a substance is rather incomplete. Spectroscopy methods, which are based on interaction of light and matter, have contributed a lot to our understanding of water. Recently, established, scientific discipline of aquaphotomics aims to integrate the knowledge these methods acquired about water based on its interaction with light, into one “omics” discipline which relates the structure of water with its functionality, and whose ultimate objective is better understanding of water as a matrix of aqueous and biologic systems [7]. In this regard, aquaphotomics have made significant novel discoveries, and utilized the properties of water in various application fields ranging from water and food quality monitoring, microbiology, to biomeasurements, biodiagnostics and biomonitoring [8,9,10]. Owing to the wide range of aqueous systems and biologic systems aquaphotomics studied, some novel, surprising insights have been discovered, which place focus on importance of water structure, hydrogen bonding and temperature.

The effect of temperature has always been one of the most studied phenomena in spectroscopy with respect to its influence on water structure. The very terms “structure-makers” and “structure-breakers” are coined to describe substances which induce changes in the water structure and consequently its spectrum, comparable to decrease in temperature and increase of temperature, respectively. Effects of salts or sugars are commonly described using those terms [11,12,13]. The known influence of temperature on hydrogen bonding in water is something that can be used as an etalon for better understanding of how substances affect the structure of water in various solutions. Among many others, principal component analysis and two-dimensional correlation spectroscopy [14] or multivariate curve resolution-alternating least squares technique [15] were used to describe the effects of temperature perturbations on the NIR spectra of water in terms of hydrogen bonding either alone or in comparison to salt perturbation.

Aquaphotomics studies made some further steps uncovering that various phenomena, related to biomolecules or functionality of living organisms and aqueous systems can be described as related to specific water molecular structure and presented using spectral pattern.

For example, in one work, concerned with the detection of UV-induced damage on DNA structure, it was found that the aqueous solutions of UVC-damaged DNA caused increase in hydrogen bonded water—i.e., that damaged DNA was a structure-making element causing changes of water similar to low temperature [16]. In another study, which explored cold tolerance ability of different soybean cultivars, it was found that the water structure in the leaves of those cultivars with higher cold tolerance ability even at low temperatures preserved the water in less-hydrogen bonded state compared to those cultivars who are more susceptible, as if the environmental temperature would have been in fact higher [17]. What these novel insights into the water structure and analogy with temperature influence provided is a novel knowledge and better understanding of the water functionality at different levels of organization of biologic systems.

One of the main visualization and analytical tools of aquaphotomics, is the so-called aquagram [8,18] which provides a comprehensible demonstration of the ratios of different water species present in a sample. Aquagrams have been found very useful in many applications, such as diagnosis of estrus in giant pandas [19], orangutans [20] and cows [21] and showing the different water spectral patterns of probiotic and non-probiotic bacteria strains [22], or for example, revealing different types of water in the soft contact lenses, in a completely nondestructive manner [23,24,25]. The cited references demonstrated the usefulness of the presentation of the water spectral patterns in aquagrams.

In order to unify the relation between water species depicted by aquagrams and related water functionalities we propose to use temperature as a common denominator to express changes of light absorbance at each of the water vibrational frequencies as changes caused by the most influential perturbation for water—temperature. Considering the advantages of better understanding of the functionality of water structure if the analogy is made with the influence of temperature, the objective of this work was to develop a novel method and a visualization tool, so called temperature-based aquagram which translates the effects of any type of perturbation of water structure in aqueous or biologic systems to the equivalent effects that would have been caused by the temperature changes and expressed in temperature units. The need to introduce the temperature-based aquagrams arose from the experiments on more complex systems, such as previously described, where it was observed that certain phenomena, (caused by solutes for example) have effects on the water molecular structure of the system and contribute to its functionality in the way analog to the changes in temperature.

**Figure 1:**
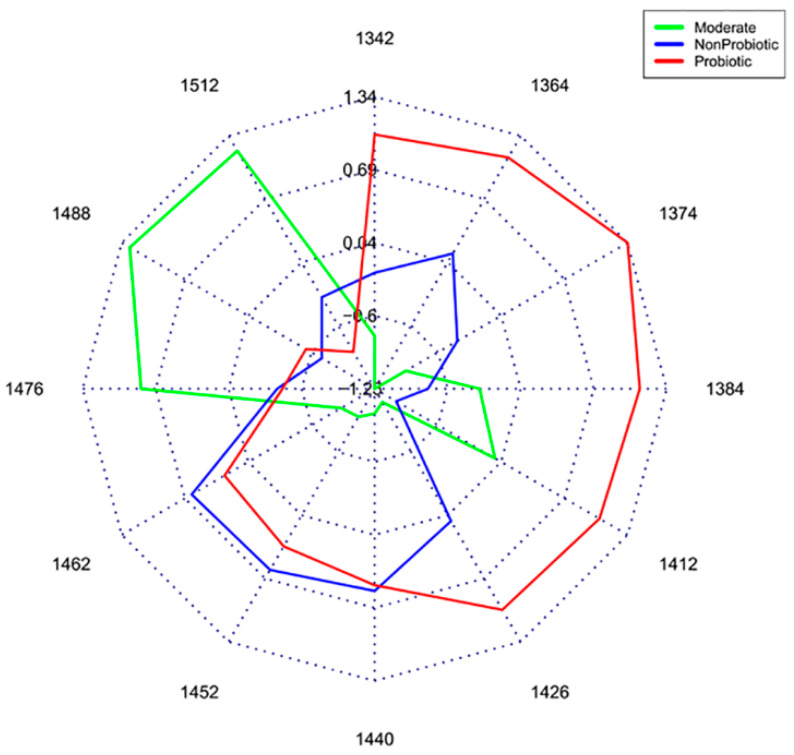
Water spectral pattern presented on aquagrams shows different water structure in different bacteria strains (Reprinted with permission from Slavchev, A., Kovacs, Z., Koshiba, H., Nagai, A., Bázár, G., Krastanov, A., Kubota, Y. and Tsenkova, R. 2015. [22]).

In a study concerned with classification of bacteria based on the probiotic strength it was found that the strong probiotic bacteria, compared to the moderately strong strains or non-probiotic strains, create less hydrogen-bonded water species (Figure 1) [22]. On the other hand, the spectra of pure water at different temperatures show shift of the main band towards the shorter wavelengths with increasing temperature [14], i.e., showing that increase in temperature results in breaking of the hydrogen bonds. If the two cases are compared, it can be seen that probiotic bacteria affect surrounding water similarly to the influence of temperature increase, as if they are a structure-breaking element [22]. This conclusion supports the novel insight into molecular mechanisms of how probiotics work–they increase solubility of substances in water [26], just like the increase in temperature of the water would contribute to better solubilization.

From this example, it can be seen that comparisons with effect of temperature can be useful and contribute to better intuitive understanding of the functionality of the studied system. Recalling the soybean cultivars example, the role of many substances that plants accumulate in response to cold stress, becomes more obvious—their function is to provide a certain water molecular structure in the leaves as if the temperature of the environment is different [17,27].

The concept of the novel visualization tool—a temperature-based aquagram—was developed similarly to the one Bernal and Fowler introduced long ago—the so called “structural temperature” concept [28]. The “structural temperature” is the respective temperature at which pure water would have effectively the same molecular structure as the water of the aqueous system under study. At the time of introduction, no adequate method that could describe that structure was found; there were propositions that it can be estimated based on the measurements of viscosity, Raman spectra or X-ray diffraction. In addition, the intended purpose was mainly concerned with the applications in analysis of electrolyte solutions, and since the concept did not offer the possibility to separate effects of the individual ions, the entire idea was more or less abandoned [29]. However, the structural temperature concept fits well into the framework of aquaphotomics, which places the water spectral pattern and the respective water functionality of the system in the central place. In contrast to “reductionistic omics methods” which are focused on isolating the biomolecules and separation of elements of the system, the aquaphotomics views each aqueous or biologic system as a whole, where all the components of the system exert their influence on the water matrix, whose structure is directly related to the function of the system [9,10]. Just like the temperature is the macroscopic, measurable characteristic, arising from the molecular structure of the system, so is the light absorbance at each water specific vibrational frequency, and one can benefit from expressing one in the terms of another.

The purpose of this study is to introduce a novel visualization tool, which expresses the effects of any type of perturbation of water molecular structure in aqueous or biologic systems to the equivalent effects that would have been caused by the temperature and expressed in temperature units. To illustrate this, we have chosen a simple salt solution and a concentration as a major perturbation. Salt is chosen as it is not near infrared active substance, hence the changes in absorbance of the solutions are only due to the changes in water molecular structure [15,30]. Following the steps provided in the study, one can easily replicate the experiment, develop the temperature-based aquagrams and use it further for specific purposes. While in this study, the analysis and the results are presented for only 1st overtone of water region, the same methodology is applicable for any region of the water absorbance spectra. This new method provides numerical results on a clearly defined scale with confidence intervals. It enables the comparison of results across time and different experiments and provides information about the statistical significance of the found differences.

## 2. Results and Discussion

The spectral data in the wavelength interval of 1300 to 1600 nm for the two experiments were separately subjected to principal component analysis (PCA). The PCA score plots demonstrated the multidimensional patterns of the spectral data. Specifically, the highest variations displayed in the first principal components (PC1) were related to temperature and concentration of potassium chloride, representing 99.3% and 99.1% of the spectral variation in case of the temperature and the potassium chloride experiments, respectively. The PCA results did not show outliers either in the temperature or the potassium chloride datasets. The raw and 2nd derivative spectra of the two experiments were analyzed separately to discover the wavelengths exhibiting the largest changes caused by the temperature and salt perturbations.

### 2.1. Results of Temperature Experiment

**Figure 2:**
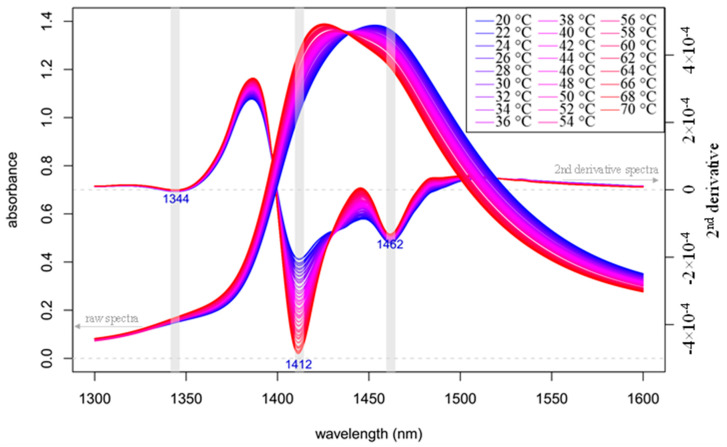
Raw and 2nd derivative (calculated with Savitzky–Golay filter using 2nd order polynomial and 21 points) absorbance (logT^−1^) spectra in the spectral range of 1300–1600 nm (OH first overtone) of Milli-Q water in the temperature range of 20–70 °C (*n* = 78).

The raw and 2nd derivative absorbance (logT^−1^) spectra in the spectral range of 1300 to 1600 nm of Milli-Q water in the temperature range of 20 to 70 °C are shown in Figure 2. The main feature of the NIR spectrum of water is a broad peak around 1450 nm, comprised of several overlapping bands, described mainly as the 1st overtone of the OH stretching vibration [31]. This observation is confirmed by the second derivative spectra which indicated very intense bands at 1412 and 1462 nm. These bands are well known as bands associated with free water molecules [32,33,34] and strongly hydrogen-bonded water [14,35], respectively.

From a spectroscopic point of view, an increase in temperature has been interpreted as a decrease of number of hydrogen bonds [14,34], while others explain it via weakening of hydrogen bonds [36]. There is agreement, regardless of one’s preferred theory, that the change of temperature causes alteration in hydrogen-bonding configurations of water as described in more details previously [37].

Figure 2 shows a “blue shift”, i.e., movement of the main band towards the shorter wavelengths with increasing temperature and an isosbestic (temperature invariant) point. These phenomena are well studied in scientific literature [14,34,38]. Evidence clearly suggest that less hydrogen-bonded water is predominant at higher temperature, while spectra acquired at low temperature are represented mostly in the more hydrogen-bonded area where water molecules form species with one (S1), two (S2), three (S3) and four (S4) hydrogen bonds [39].

**Figure 3:**
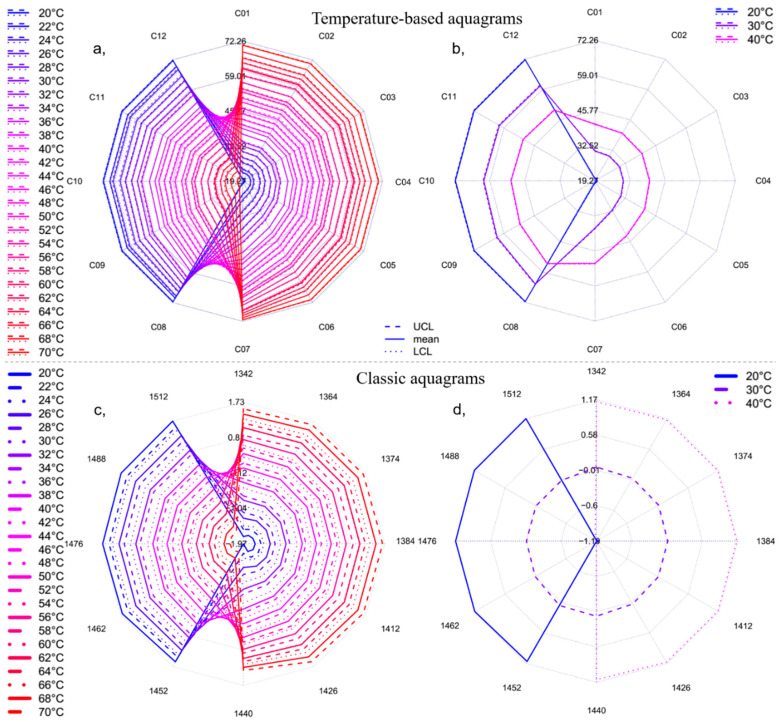
Aquagrams of Milli-Q water in the temperature range of 20–70 °C, (**a**,**b**) with 95% confidence intervals calculated with temperature-based aquagram calculation method, (**c**,**d**) calculated with the classic calculation method, (**a**,**c**) all the 26 temperature steps (*n* = 78) and (**b**,**d**) on three selected temperature steps (*n* = 9) to show the stability of the methods (UCL—upper confidence level, LCL—lower confidence level).

Despite the competing theories about the water structure, aquaphotomics identifies water-specific absorbance bands with higher variations caused by respective perturbation for the system of interest and uses them to depict the unique spectral pattern as an integrated marker of the system–perturbation interaction. The presentation of the 12 specific water coordinates [7] of the spectra of Milli-Q water acquired in the temperature range of 20 to 70 °C together with the 95% confidence intervals is given in Figure 3a,b, calculated with the temperature-based aquagram calculation method. In the case of working with more complex system, it would be advisable to follow the protocol of aquaphotomics analysis which can provide more thorough examination of activated water absorbance bands and not necessarily limit the presentation to only the 12 coordinates as is chosen here. The aquagram provides an easy-to-comprehend presentation of the phenomena described above, i.e., the change of the strongly and weakly hydrogen-bonded patterns, based on Figure 2. The movement of the higher absorbance towards shorter wavelengths with increasing temperature is convincing. The scales of the aquagrams (Figure 3a,b) express the effect of perturbation occurring at the 12 coordinates (i.e., in the defined wavelength ranges) in degrees Celsius equivalent. Therefore, in this specific example the radial values show the temperatures corresponding to the signal acquisitions (20 to 70 °C). The plotted dashed and dotted lines represent the upper and lower confidence levels of the aquagram values, respectively, for each single analyzed temperature step. There was no overlapping of the confidence intervals (95%) of the Milli-Q samples measured at different temperature observed, meaning that 2 °C temperature changes caused statistically significant effects on the individual coordinates (i.e., in the defined wavelength ranges). The stability of the temperature-based aquagram calculation is presented in Figure 3b, by depicting only three selected temperature levels (20, 30 and 40 °C). Though the spectral dataset used to calculate the temperature-based aquagram of Figure 3b is only a subset of the dataset used to calculate Figure 3a, the shape and the range are the same in both cases. This type of stability was not possible with the “classic” aquagram (Figure 3c,d). These examples present the applicability and the additional benefits of the newly developed temperature-based aquagram for the evaluation and presentation of the water species in the 12 coordinates (representing defined wavelength ranges) through the evaluation of a well-known perturbation, i.e., the effect of temperature on the water spectral changes.

### 2.2. Results of Potassium Chloride Experiment

**Figure 4:**
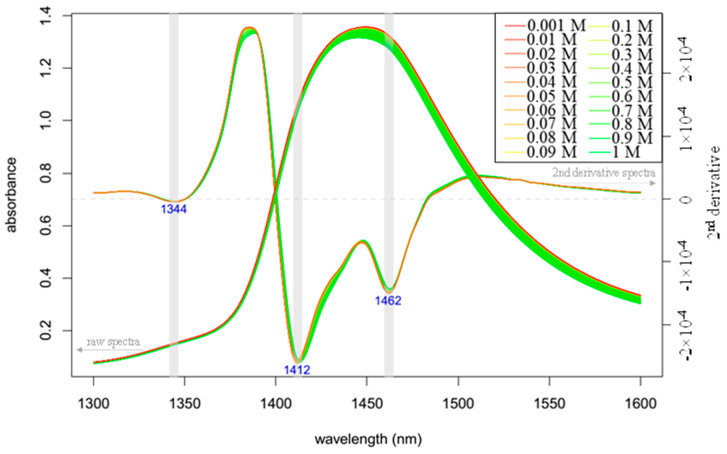
Raw and 2nd derivative (calculated with Savitzky–Golay filter using 2nd order polynomial and 21 points) absorbance (logT^−1^) spectra in the range of 1300–1600 nm (OH first overtone) of 0.001–1 M KCl solutions (*n* = 180).

The raw and 2nd derivative absorbance (logT^−1^) spectra of the aqueous solutions of 0.001 to 1 M potassium chloride salt in the 1300–1600 nm wavelength range (OH first overtone) are presented in Figure 4. The main component of the aqueous solution, other than water, is KCl, which has no absorption in the NIR region; thus, it is not surprising that the spectra show a broad peak around 1450 nm. The second derivative spectra also provide similar patterns to those of the Milli-Q water acquired at different temperature, indicating bands at 1412 and 1462 nm. The trend of the shift in the peak position was similar to that observed in the temperature-perturbed pure water (i.e., the peak moves towards lower wavelengths as temperature is increased), but the actual peak locations were different from those for the pure water. The effect of low concentrations of salts diluted in water has been illustrated by the changes in the OH bonding of water molecular systems in many experiments [15,40,41,42]. As salts do not absorb NIR light, accurate measurement of even low concentration of salts, published in the above-mentioned papers, means salts change the surrounding water molecular structure according to the number of the solvent molecules in the solution which still can restructure the water molecular system.

This phenomena is not new; Bernal and Fowler [28] showed that the addition of electrolytes changes the spectrum of water in the infrared overtone region. Lin and Brown [43] also analyzed the effects of different salts on water spectra, and the authors were able to build accurate regression models using the spectral range of 1490 to 1610 nm for salt content prediction.

The spectra shown in Figure 4 imply that the increasing concentration of KCl causes “blue shift”, i.e., a shift towards the spectral range referring to the band of less hydrogen-bonded water molecules. Our findings, hence show a good agreement with the results of previous research [15,40,41,42].

**Figure 5:**
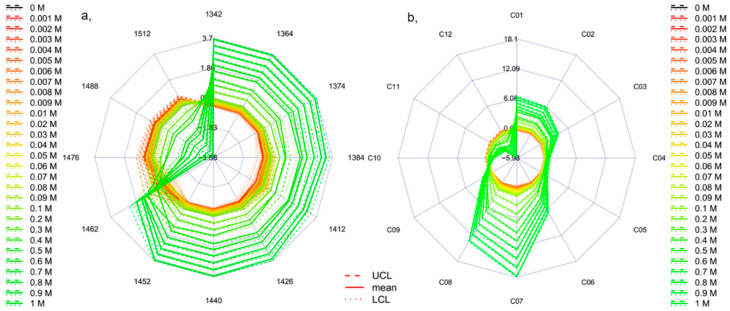
Aquagrams with 95% confidence intervals of Milli-Q water (*n* = 150) and 0.001–1 M KCl solutions (*n* = 180) calculated with the classic (**a**) or the temperature based aquagram (**b**) calculation methods on the individual concentrations (UCL—upper confidence level, LCL—lower confidence level).

A more detailed evaluation of the proposed 12 specific water coordinates [7] of the spectra of the aqueous solutions of potassium chloride compared to the spectra of water is provided in Figure 5. The plots show the aquagrams of the single concentration levels together with the respective 95% confidence intervals calculated with the “classic” method (Figure 5a) and the new temperature-based aquagram calculation method (Figure 5b). The relative position of the patterns of the single concentration levels shows some similarity for the two methods. Furthermore, in both methods, spectral patterns of the highest concentration range (100–1000 mM) show the highest difference from the spectra of the lower ranges and Milli-Q samples (the latter one in black). This pattern explains the same phenomena which were found based on the raw spectra, i.e., a higher concentration of salt create water with less hydrogen bonds and decreases the number of water species to more hydrogen bonds. For the higher concentration ranges the aquagrams of the individual concentrations do not overlap, i.e., addition of 0.01 or 0.1 M KCl caused statistically significant change at the individual coordinates.

However, the results also show an increasing tendency of the C7 coordinate (1432–1444 nm) with increasing concentration of KCl, which can be assigned to water molecules with one hydrogen bond (S1), meaning that increase in concentration of salt leads to increase in the number of these molecules [39].

**Figure 6:**
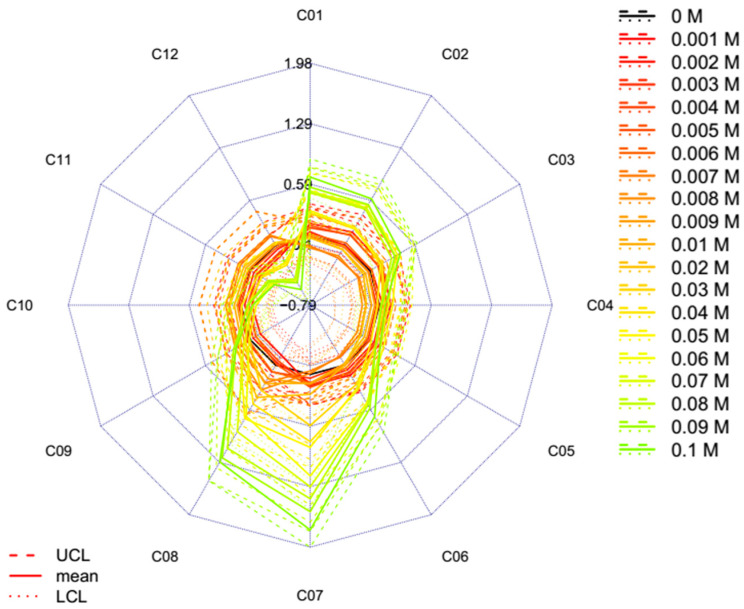
Aquagrams with 95% confidence intervals of Milli-Q water (*n* = 150) and 0.001–0.1 M KCl solutions (*n* = 120) calculated with the temperature based aquagram calculation method on the individual concentrations (UCL—upper confidence level, LCL—lower confidence level).

The dominantly higher effect of the higher concentrations on the spectral pattern hides the pattern of the lower concentrations; therefore, the calculation of the aquagrams were performed using the spectral data set for the two lowest concentration ranges only (Figure 6). The phenomena of the higher concentrations on the water spectral pattern, namely the higher concentration being more dominantly a structure breaker, can be observed in case of the concentration of samples at the concentration range of 10 to 100 mM. It is interesting to note the similarities to the pattern of the aquagrams calculated on all the three ranges (Figure 5b).

The aquagrams of the higher concentrations (10–100 mM) show consistency with the previous findings regarding the structure-breaking characteristic as displayed in Figure 6. More specifically, the higher concentration samples present higher absorbance values in the range between 1342 and 1374 nm, i.e., C01-C03 that refer to the 1st overtone of free OH stretch (OH–(H_2_O)*_n_*, *n* = 1…4) [44,45] and 1440 and 1452 nm, i.e., C07-C08 that are known as the bands of water hydration [35] and water molecule connected to another water molecule (S_1_) [14,46] and the symmetric and asymmetric stretching of first overtone of water [35,46,47]. However, in the range between 1476 and 1512 nm, i.e., C10-C12, the higher concentration samples show lower values and these wavelengths are usually assigned to strongly hydrogen bonded water [14,35] and aqueous protons ([H^+^•(H_2_O)_6_]–H_2_O in H_5_O_2_^+^ symmetric stretch) [48]. These findings also mean decreasing concentration of salt causes a shift towards longer wavelengths, i.e., the range referring to the band of more hydrogen bonded water molecules.

Using the additional benefit of the temperature-based aquagram calculation method, further results can be achieved from Figure 6. The addition of, for instance, 0.1 M potassium chloride to Milli-Q water results in water structural changes equivalent to changes caused by temperature of about 0.65, 0.6, 0.3, 0.1, 0.2 0.6, 1.8, 1.2, 0.2, −0.1, −0.3 and −0.6 °C at C01, C02, C03, C04, C05, C06, C07, C08, C09, C10, C11 and C12 coordinates, respectively. Furthermore, having calculated the confidence intervals, the statistical significance of the differences is also available. For example, calculations showed that addition of 0.02 M KCl to Milli-Q caused change equivalent to the change due to 0.33 and 0.23 °C temperature increase at coordinates C07 and C08 when compared to pure Milli-Q, which was found significant (*p* = 0.05), too.

**Figure 7:**
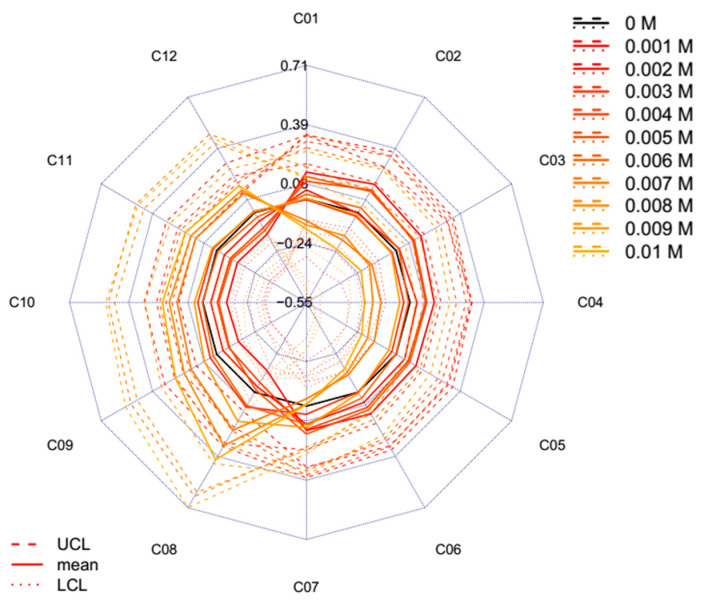
Aquagrams with 95% confidence intervals of Milli-Q water (*n* = 150) and 0.001–0.01 M KCl solutions (*n* = 60) calculated with the temperature based aquagram calculation method on the individual concentrations (UCL—upper confidence level, LCL—lower confidence level).

The aquagrams of the lowest concentration range (1–10 mM) calculated with the temperature-based aquagram method is shown in Figure 7 to further evaluate the findings of the effects of decreasing concentrations of salt. The results suggest that we can observe concentration levels of 5 to 10 mM that cause a shift towards longer wavelengths, i.e., the range referring to the band of more hydrogen-bonded water molecules compared to the spectrum of Milli-Q water. These findings imply that salts can also have structure-breaker and structure-maker effects on water structure similar to the behavior of sugars [13]. However, the aquagrams of the even lower concentrations (1 to 5 mM) also show alteration with nearly the same deviation, but towards shorter wavelengths. This may suggest that changing between structure-breaker and structure-maker properties of salt exist at such a low concentration, but the discernment of these minor changes would require very accurate measurements.

## 3. Materials and Methods

### 3.1. Samples

Two experiments with different sources of perturbations were conducted to demonstrate the procedure of temperature-based aquagram development and show the advantages in comparison to the representation of spectral data using classic aquagrams. In both experiments, pure Milli-Q water was used as a sample (Milli-Q purification system (Millipore, Molsheim, France, resistance = 18 MΩ) and in the first experiment perturbation of the sample was caused by changes in temperature, while in the second one, by changes in the concentration of salt.

### 3.2. The Temperature Experiment

The effect of temperature perturbation on water near-infrared spectra has been well-studied and is thoroughly described in the literature [14,34,49]. Therefore, the experiment was performed on Milli-Q water in the temperature range of 20 to 70 °C to acquire spectra which could be used for the evaluation of the aquagram methods. The Milli-Q water was produced by a Milli-Q purification system (Millipore, Molsheim, France, resistance = 18 MΩ). The spectral acquisition was performed at 2 °C increments, resulting in 26 temperature steps in the range of 20 to 70 °C.

### 3.3. The Potassium Chloride Experiment

The addition of salt to water at different concentrations is also an often evaluated perturbation [42,50]. Therefore, an experiment was performed with different concentrations of aqueous solutions of potassium chloride. Potassium chloride (KCl, M = 74.56 g mol^−1^, purity min. 99.0% mass/mass) was purchased from Wako Pure Chemical Industries, Ltd. (Kobe, Japan). Aqueous solutions were prepared in different concentrations of KCl, in the range of 1 to 1000 mM. Three concentration ranges were prepared: Range A, from 100 to 1000 mM concentration, in steps of 100 mM; Range B from 10 to 100 mM, in steps of 10 mM and finally, Range C, in 1 to 10 mM in 1 mM concentration steps. Each dilution was prepared by serial dilution from the stock samples with the highest concentration in a given range (A, B or C) and prepared in two replicates, resulting in two independently prepared sets of samples. Stock solutions were prepared and further serially diluted with added Milli-Q water step-by-step to reach the appropriate concentrations—a solution created in each step was further diluted to prepare the next lower concentration.

### 3.4. NIR Spectral Acquisition

A FOSS-XDS spectrometer (FOSS NIRSystems, Inc., Hoganas, Sweden) equipped with a Rapid Liquid Analyzer module including a temperature-controlled 1 mm pathlength cuvette holder was used to measure transmittance spectra (logT^−1^) of the Milli-Q samples for the temperature experiment and of the aqueous solutions for the KCl experiment. Spectral acquisition was performed by saving three consecutive spectra in the range of 400–2500 nm at 0.5 nm spectral steps. Each saved spectrum was the average of 32 successive scans.

A thermal bath with continuous water circulation was attached to the Rapid Liquid Analyzer module to ensure the required temperature of the sample during scanning in the range of 20 to 70 °C at 2 °C increments.

The same apparatus was used to provide a constant temperature of 28 °C where each aqueous solution of potassium chloride was incubated for 90 s to equilibrate to the required temperature before scanning. Milli-Q water samples were measured as every fifth sample during the KCl experiment to provide environmental controls.

The total number of spectra for the temperature experiment was 78 (26 temperature steps × 3 consecutive scans) and for the KCl experiment was 330 (30 concentrations × 2 repeats × 3 consecutive scans + 150 Milli-Q control scans) (Appendix A Dataset).

The FOSS-XDS instrument was operated using VISION 3.5 software (FOSS NIRSystems, Inc., Hoganas, Sweden). In both experiments, reference spectra were recorded before every sample.

### 3.5. Statistical Data Analysis

Only the wavelength interval of 1300 to 1600 nm, corresponding to the first overtone of the O–H stretching band [51] was used for the evaluations. For the purpose of explaining methodology of how to develop temperature-based aquagrams, in this study, the focus is placed on this particular part of the water absorbance spectra, because it is best understood so far in the terms of the water molecular species whose absorbance bands are well-resolved and their assignments known [7]. In the first overtone of water as a result of systematization of experimental work done on many different systems, not only different aqueous solutions, but also a great variety of biologic systems under different perturbations 12 water absorbance bands termed WAMACs (Water Matrix Coordinates)–each range from 6 to 12 nm width, were discovered [7]. The great body of evidences in scientific literature provided the meaning to these ranges—i.e., it was possible to connect each of these 12 coordinates to specific water molecular species. In aquaphotomics studies the 12 WAMACs are called, coordinates because they represent windows in the spectra through which water structure of the system under study can be observed. In this study, the 12 WAMACs will be used to develop aquagrams, but it should be noted that the methodology explained is applicable for any region of the water absorbance spectra; it is not necessarily limited to the coordinates chosen here. Here, we chose for the reason of simplicity and because they are well-understood to use only those. However, depending on the system under study and the range of the spectra used, the reader is advised to follow the aquaphotomics protocol of analysis for extraction of the “activated water absorbance bands–WAMACs [8] and then follow the further instructions to develop temperature-based aquagrams as described below.

Before the development of aquagrams, exploratory analysis was performed. First, Principal component analysis (PCA) [52] was used to describe multidimensional patterns in the spectral data and to discover outliers. The raw and 2nd derivative spectra were plotted to visualize the spectral changes induced by temperature perturbation and by the perturbation of salt concentration on the spectra of Milli-Q water and aqueous solutions of potassium chloride, respectively. The 2nd derivative spectra were calculated using a Savitzky–Golay filter [53] using the 2nd order polynomial and 21 points.

#### 3.5.1. Calculation Protocol of “Classic” Aquagram

The absorbance values at specific water matrix coordinates (WAMACs) [7] define the water spectral pattern (WASP), which is different for different perturbations. The WASP can be visualized by a chart called the aquagram [18,19]. This representation of the WAMACs was first introduced by Tsenkova [18]. This aquagram (from now on called the “classic” aquagram) displays the multiplicative-scatter-corrected (MSC) (or standard-normal-variate (SNV)) transformed, normalized and averaged absorbance values of different samples or sample groups at 12 specific characteristic wavelengths. As, mentioned, these specific wavelengths were experimentally discovered as absorbance bands of specific water molecular species in previous studies and are later confirmed by overtone calculations of already reported water absorbance bands in the infrared range [7]. The aforementioned water absorbance bands cover various form of water molecular species and are thus useful to depict characteristic spectral patterns in the first overtone region of water. This calculation can be summarized by Equation (1).

(1) $$A_{\lambda }^{\prime }=\frac{A_{\lambda }-\mu_{\lambda }}{\sigma_{\lambda }}$$

where, $A_{\lambda }^{\prime }$—value on aquagram for a given wavelength; *A_λ_*—absorbance after MSC applied on 1st overtone region of OH (i.e., 1300–1600 nm); *μ_λ_*—mean of all spectra for the examined group at a given wavelength (after MSC applied); *σ_λ_*—SD of all spectra for the examined group at a given wavelength (after MSC applied); *λ*—12 wavelengths (1342, 1364, 1374, 1384, 1412, 1426, 1440, 1452, 1462, 1476, 1488, 1512 nm) [7].

The classic aquagram shows the relative fingerprint, i.e., the WASPs, in the context of all spectra in the examined group [18,19,21,54].

Recently, this aquagram calculation method was extended by adding the possibility to observe the statistical significance of the differences presented on the aquagrams. Therefore, besides the average spectra of the individual groups used to plot the aquagrams, the respective confidence intervals are also calculated using the so-called Bootstrap method [55]. This improvement makes it possible to plot the aquagrams together with their upper and lower 95% confidence interval limits.

#### 3.5.2. Calculation Protocol for Newly Developed (Temperature-Based) Aquagram

The newly developed temperature-based aquagram calculation algorithm presents the respective water matrix coordinates in units equivalent to change in temperature and includes the respective confidence intervals. Therefore, it gives rise to the possibility to compare the WAMACs across time and also across different experiments and it provides information about the statistical significance of the differences.

**Figure A1:**
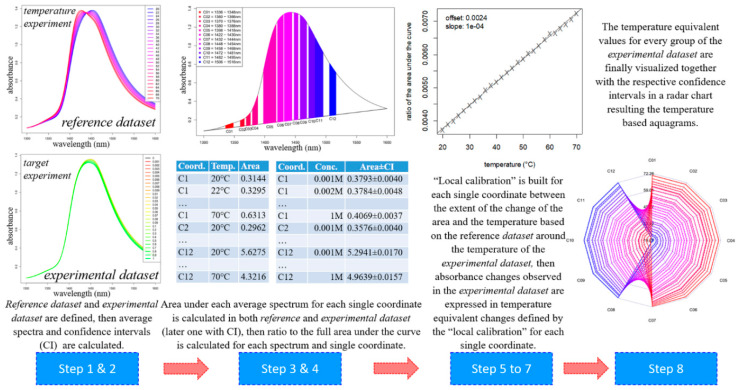
Workflow of the calculation protocol of temperature-based aquagram.

The new calculation method is based on the comparison of the areas (under the ranges of the above-mentioned 12 specific water coordinates [7]) of the respective test sample spectra and the spectra of Milli-Q water. This method aims to express the spectral pattern changes in units equivalent to the change of temperature that would cause the observed change. The calculation of this aquagram concept (from now called the temperature-based aquagram) can be summarized in the following steps. Note that the calculation steps are explained for one coordinate (C01 representing spectral range between 1336 and 1348 nm, as an example) out of the 12 coordinates (C01-C12, i.e., defined wavelength ranges) to give an easily understandable description, but the same steps have to be repeated for each of the 12 coordinates. The main calculation steps are summarized in a chart to provide an overview of the developed method (Appendix B Figure A1).

**Figure 8:**
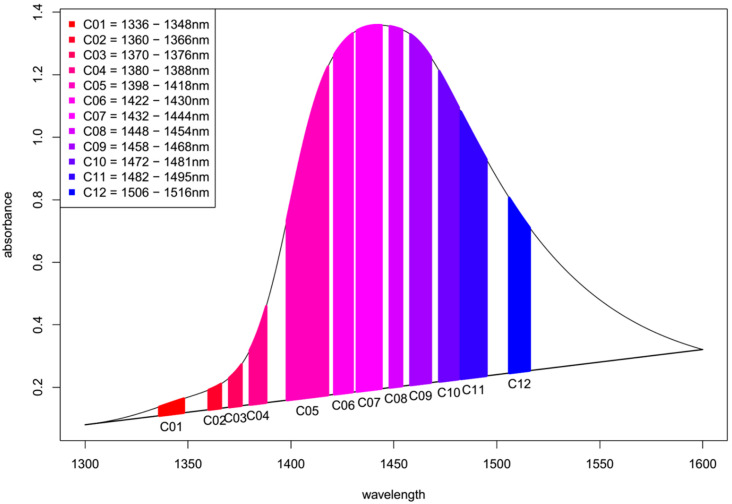
Scheme of the calculation for Area under the curve (AUC) aquagram method. Spectrum of pure water with highlighted subranges of the 12 specific water matrix coordinates (WAMACs).

Step 1. The dataset of the temperature experiment (i.e., the spectra of the Milli-Q water samples acquired during the temperature experiment in the temperature range between 20–70 °C) is defined as the reference dataset.
  The dataset of the experiment of interest is defined as the experimental dataset.
Step 2. The average spectra of the consecutive scans are calculated for each temperature step, yielding 26 single, unique spectra in the reference dataset.
  The average spectra of the groups of interest (in this case, salt concentration levels) are calculated in the experimental dataset together with their respective confidence intervals using the Bootstrap method [55], yielding as many single, unique spectra as there are groups are in the experimental dataset (plus their upper and lower 95% confidence interval limits).
Step 3. The area under the spectrum for every single average spectrum—in the reference dataset and in the experimental dataset—at the wavelength range of 1336 to 1348 nm (C01) is calculated taking into account the baseline estimated by linear fitting on the two edges of the first overtone region (i.e., 1300 and 1600 nm). In case of the experimental dataset, the areas of the respective confidence interval limits are also calculated in addition to the area of the average spectrum. Figure 8 provides graphical interpretation of the relevant areas and the wavelength regions used for the 12 coordinates.
Step 4. The ratio of the area under the curve for each single coordinate is calculated with respect to the full area under the curve for the first overtone OH region (i.e., the area of C01 is divided by the full area under the spectrum in the range of 1300 to 1600 nm). This is done for every single average spectrum, in the reference dataset and the experimental dataset (together with the respective confidence interval limits for the experimental dataset). This calculation step provides normalized values and avoids possible differences due to scattering and/or pathlength effects.
Step 5. Based on the reference dataset, a continuous array of values for the relative area of C01 (as calculated in Step 1) is calculated for a continuous temperature range from 20 to 70 °C using local polynomial regression. This is an essential step in order to accommodate the data from an experiment performed at specific temperature—see Step 6.
Step 6. The basic principle of the temperature-based aquagram method is to compare the effect of the perturbation used on the system under study which resulted in a certain water spectral pattern to the effect the temperature changes would induce in pure water. Thus, any perturbation can be expressed as an equivalent temperature effect on a Milli-Q water sample. It is necessary to perform a “local calibration” with the reference dataset around the temperature of the experimental dataset. Therefore, in this step, the temperature calibration range is defined. This range is used to express the effect of perturbation in degrees Celsius equivalent. For this, a symmetrical scale is defined from the reference dataset (calculated at Step 5) using two degrees, plus and minus around the temperature of the experiment (hence, a span of 4 °C). For example, if the experiment was performed at 25.0 °C, then the calibration range of 23.0 to 27.0 °C would be used.
Step 7. The temperature calibration equation, the relationship between the change of the temperature and change of the area of C01 at the temperature of the experiment, is determined based on the calculation performed in Step 5 on the reference dataset. (It is known how the area of C01 changes as a function of temperature described by a linear function). Therefore, it is easy to compare the changes for areas for C01 for the experimental dataset (calculated at Step 3) to the changes of the area of C01 caused by temperature, i.e., to express the changes in C01 in units of temperature (degrees Celsius) equivalent.
Step 8. The calculated temperature (degrees Celsius) equivalent value for every group of the experimental dataset is finally visualized together with the respective 95% confidence intervals in a radar chart, where the units of the axes are in degrees Celsius.
Step 9. The calculation and visualization of the results were performed using the R programing language [56,57].

## 4. Conclusions

Recently, aquaphotomics has been introduced to focus on water as a key component for providing information about the function of the entire system. Non-destructive NIR spectroscopy and aquaphotomics have been applied to explain new phenomena in the field of life sciences. In contrast to reductionistic methods in which biomolecules and other elements are analyzed separately from the system, aquaphotomics studies the aqueous systems through its water matrix.

In the present study, a newly developed temperature-based aquagram calculation method is presented as an additional tool to express the changes of water molecular structure in aqueous systems which are caused by perturbations different from temperature. Although the need to introduce temperature-based aquagrams originated from experiments on complex systems, the successful application of the new aquagram calculation method was demonstrated through the evaluation of the results of well-known phenomena. The results of temperature and salt perturbations on Milli-Q water are demonstrated in the present study.

The effect of temperature on the spectral pattern of Milli-Q water acquired in the temperature range of 20 to 70 °C is presented with the temperature-based aquagram calculation method. The method provides the presentation of the phenomena demonstrating blue shift in the first OH overtone range with increasing temperature. Furthermore, the scale of the aquagram expressed the effect of perturbation in degrees Celsius equivalent.

Aqueous solutions of potassium chloride were chosen for the experiment as KCl has no absorption in the NIR region. Thus, its effect on water spectral patterns can be clearly evaluated and presented as caused by temperature changes at respective wavelengths. Furthermore, it demonstrates that the method is invariably applicable for evaluation the effects of all types of solutes. The new temperature-based aquagram calculation method provided further information about the magnitude of the change. In other words, the results were displayed on a degree-Celsius scale that showed how much a given sample would have needed to have been warmed up or cooled down in each of the single coordinates (C01 to C12) to achieve the same results as the actual measurement, while all measurements were performed at precisely the same temperature.

Adding 0.1-M potassium chloride to Milli-Q water resulted in structural changes equivalent to an approximately 0.6 °C temperature increase in the less hydrogen-bonded, and 0.3 °C temperature decrease in the more hydrogen-bonded areas of the OH first overtone spectral region.

The examples presented here confirm the applicability and the additional benefits of the temperature-based aquagram calculation method. They provide a demonstration of the ratio of the different water species existing in different aqueous and biologic systems. Additionally, this new type of aquagram calculation displayed the spectral patterns in a meaningful scale and stable pattern independent of any modification of the evaluated dataset, which gives rise to the opportunity to compare results not only within a single chart, but also across time and different experiments.

This newly developed tool is especially suitable for visualizing water structure evolution and phase transitions, for example, in the food preservation industry, pharmaceutical development, material science and related applications.

The presented new chemometric tool, developed in R-Project, is freely accessible as an R-package from GitHub repository [58] and can be used in various fields of NIR spectroscopy and water research.

## Appendix A

Dataset. Raw spectral data used for the calculations presented in this study. Both data of temperature experiment and potassium chloride experiment is included in this dataset where the column experiment describes which spectra belong to which experiment.
